# Modulating brain networks associated with cognitive deficits in Parkinson’s disease

**DOI:** 10.1186/s10020-021-00284-5

**Published:** 2021-03-10

**Authors:** Iman Beheshti, Ji Hyun Ko

**Affiliations:** 1 Department of Human Anatomy and Cell Science, University of Manitoba, 130-745 Bannatyne Ave., Winnipeg, MB R3E 0J9 Canada; 2 Kleysen Institute for Advanced Medicine, Health Science Centre, Winnipeg, MB Canada; 3 Graduate Program in Biomedical Engineering, University of Manitoba, Winnipeg, MB Canada

**Keywords:** Transcranial direct current stimulation, Parkinson’s disease, Cognition, Positron emission tomography, Functional magnetic resonance imaging, Network analysis, Graph theory, Mild cognitive impairment

## Abstract

Parkinson’s disease (PD) is a relatively well characterised neurological disorder that primarily affects motor and cognitive functions. This paper reviews on how transcranial direct current stimulation (tDCS) can be used to modulate brain networks associated with cognitive deficits in PD. We first provide an overview of brain network abnormalities in PD, by introducing the brain network modulation approaches such as pharmacological interventions and brain stimulation techniques. We then present the potential underlying mechanisms of tDCS technique, and specifically highlight how tDCS can be applied to modulate brain network abnormality associated with cognitive dysfunction among PD patients. More importantly, we address the limitations of existing studies and suggest possible future directions, with the aim of helping researchers to further develop the use of tDCS technique in clinical settings.

## Background

Parkinson's disease (PD) is the second most common of neurodegenerative disorders, and it is characterized by the symptoms bradykinesia, akinesia, resting tremor, impaired posture and balance, gait disturbance, and loss of automatic movements (Jankovic 2008). Almost all PD patients experience non-motor symptoms and non‐motor fluctuations over time (Schapira et al. 2017). Brain imaging techniques such as functional magnetic resonance imaging (fMRI) have provided useful insight to PD and, owing to recent technological advances, these methods enable us to have a better understanding of the impact of PD in the brain.

Neuroimaging studies have shown the association between parkinsonian motor symptoms and various neurophysiological changes. For example, putamen dopaminergic degeneration (e.g., decreased dopamine transporter binding) (Antonini et al. 1995), and spatial pattern of cortical and subcortical glucose metabolism (e.g., decreased metabolism in the premotor and parieto-occipital regions and increased metabolism in the putamen, pallidum, thalamus, pons, cerebellum and sensorimotor area) (Eidelberg et al. 1994). Grey matter volume reductions in the striatum as well as in the extrastriatal regions including temporal lobe, amygdala, angular gyrus, middle occipital gyrus, fusiform gyrus, superior frontal gyrus, anterior cingulate, and insula have been consistently reported in voxel-based morphometry studies in PD (for meta-analysis, see Xu et al. 2020). Parkinsonian cognitive impairment has been attributed to changes such as caudate dopaminergic degeneration (Schrag et al. 2017), decreased glucose metabolism in prefrontal regions (Marié et al. 1995), hippocampal atrophy (Camicioli et al. 2003), and white matter diffusion changes (e.g., abnormal diffusion tensor imaging variables in the bilateral frontal, parietal, temporal regions, and hippocampus) (Zhang and Burock 2020). The advances in network analytic approaches have revealed functional abnormalities corresponding to patients’ cognitive impairment. Resting-state fMRI and positron emission tomography (PET) elucidate these changes by showing where the spatial brain networks are defined by synchronous fluctuation of fMRI signal over time (Baggio et al. 2014), and group-wise variation across each individual’s glucose metabolic pattern (Huang et al. 2007b), respectively. Interestingly, the fMRI-based PD-specific networks and FDG-PET-based evidence were topographically similar (Vo et al. 2017). The normalization of abnormal brain network expression has been associated with symptom amelioration in PD patients (Schindlbeck and Eidelberg 2018), and modulating abnormal brain network organization has been proposed as an alternative objective for novel treatment development (Wolters et al. 2019).

In general, brain network modulation methods for PD can be categorized into two main groups.

1. Pharmacological interventions, such as levodopa and dopamine agonists, are typically tailored to address motor symptoms. The effects of dopamine replacement therapy on PD cognition are somewhat controversial such that it improves certain domain of cognition (e.g., executive function) while impairing impulse control, which seeded the inverted-U shape hypothesis—dopamine therapy dose that is optimized for motor symptom amelioration may be overdosing the ventral striatum where the dopaminergic innervation is relatively spared in PD (Cools et al. 2019). Recent epidemiological studies suggested that the pathologies of cognitive deficits found in PD can be further subtyped into two categories, dopaminergic vs. non-dopaminergic (Kehagia et al. 2013). The dopaminergic degeneration is often associated with executive function deficits and fronto-striatal circuit abnormality, and is responsible for mild cognitive impairment. These symptoms generally respond well to dopamine replacement therapy. On the other hand, the attentional and visuospatial deficits are dopamine-independent, and are associated with more posterior brain. The presence of these symptoms is a risk factor for later development of dementia. These non-dopaminergic cognitive symptoms tend to respond to cholinesterase inhibitors, which are often prescribed to treat symptoms associated with Alzheimer’s disease (AD) (Mamikonyan et al. 2015; Svenningsson et al. 2012). Nevertheless, the underlying mechanisms of neither dopaminergic therapies nor anti-AD therapies are fully understood and their clinical benefits are limited.
2. Brain stimulation techniques use electricity to facilitate or suppress/inhibit specific regional brain activities, and can be categorized into invasive and non-invasive approaches. The most common form of invasive treatment is deep brain stimulation (DBS) in which electricity can be supplied directly by electrodes implanted in the subcortical area. DBS technique has been widely used for improving motor symptoms (Fasano et al. 2012; Piper et al. 2005; Roper et al. 2016).

However earlier studies reported worsening cognitive symptoms after DBS and thus patients with cognitive deficits are not recommended to receive DBS (Bronstein et al. 2011; Mehanna et al. 2017). Transcranial magnetic stimulation (TMS) and transcranial direct currention stimulation (tDCS) are the two most common non-invasive brain modulation techniques. Traditionally, TMS has been a more favored research tool, owing to its focality of stimulation. A magnetic coil is held over the anatomical target region, and the brain area affected by TMS-induced over threshold eddy current is estimated to be 1–2 cm^2^. Conventional tDCS lacks this focality. Typically, two saline-soaked sponges (12–20 cm^2^) and tDCS electrodes are secured on the scalp over the targeted brain region and on the control site (often arbitrarily chosen, e.g., contralateral supraorbital area). A very large expanse of brain regions in between the two sponges are therefore affected indiscriminately. As a result, more extensive literature reviews exist for the use of TMS in network modulation of Parkinson’s disease (Hallett 2007; Ko et al. 2013), but lesser is known for tDCS. As of November 17, 2020, a PubMed search for “transcranial magnetic stimulation Parkinson imaging network” returns 12 review articles, while a search for “transcranial direct current stimulation Parkinson imaging network” returns 3 review articles.

In this review, we focused on the application of tCDS for restoration of brain network abnormality associated with cognitive deficits in PD. The safety of tDCS has been assessed and documented elsewhere (Bikson et al. 2016). The clinical efficiency of tDCS has been successfully assessed on different neurological conditions, such as AD and mild cognitive impairment (MCI) (Holczer et al. 2020), attention-deficit hyperactivity disorder (Salehinejad et al. 2020), depression (Palm et al. 2016), and PD motor symptoms (Manenti et al. 2018; Lee et al. 2019), as well as in healthy working memory (Mancuso et al. 2016). The structure of this review is split into three sections. First, we provide a brief overview of brain metabolic network abnormalities in PD. Next, we introduce the potential underlying mechanisms of tDCS. Then, we present a review of the publications that used tDCS to improve the cognitive dysfunction in PD. Finally, we address the limitations of existing studies and suggest potential future paths for therapeutic tDCS trials for PD cognitive deficits.

## Brain network abnormalities in PD

Network analysis is a relatively recently popularized approach in investigating functional brain imaging data for circuit abnormalities in neurodegenerative diseases. Moeller and colleagues have developed a unique strategy, scaled subprofile modeling (SSM), to build spatially distributed brain networks on the basis of sources of variability coupled by principal component analysis (PCA) technique (Alexander and Moeller 1994; Moeller and Strother 1991). SSM/PCA has been widely used in the investigations of spatial covariance patterns corresponding with specific disease states using different functional brain imaging techniques (Carbon et al. 2003; Habeck et al. 2005; Huang et al. 2007a). For example, spatial covariance analysis through resting-state FDG-PET images would allow us to uncover the regional metabolic variations corresponding to motor and cognitive symptoms among PD individuals (Eidelberg 2009).

PCA is a widely used signal processing technique that reduces the dimensionality of data without much loss of information. PCA identifies principal components (PCs) and ranks them according to the variances accounted for (VAF). For instance, if VAF for a PC is less 5%, it may be neglected. In brief, PCs are eigenvectors resulted from singular value decomposition of a covariance matrix (region × region across all subjects). And thus, if two distinct groups (e.g., disease vs. control) are pooled and if the disease sufficiently affects the overall brain metabolic pattern, SSM/PCA can characterize the disease-related brain metabolic covariance pattern that differentiates the two groups (Spetsieris et al. 2013). Typically, a sample size of 10–30 individuals per group is sufficient to produce a stable disease-related brain FDG-PET covariance pattern (Ma et al. 2007).

The SSM/PCA approach does not only capture the most discriminative features between groups—it enables one to quantify how much an individual’s brain metabolic pattern resembles a pathological brain metabolic pattern. The resulting subject scores indicate how greatly each individual expresses this disease-related configuration (i.e., how much an individual brain “looks like” a classic PD brain). The well characterised PD-related metabolic covariance pattern (PDRP) is the first PC resulted from the SSM/PCA on pooled group of 33 PD patients and 33 age-matched healthy control (Ma et al. 2007). The PDRP has been spatially characterized by increased metabolic activity in the pallidum, thalamus, pons, and cerebellum, accompanied with decreased metabolism in the premotor cortex, supplementary motor area (SMA), and parietal areas (Ma et al. 2007). The subject scores of PDRP have been used as an informative biomarker/measurement for monitoring/tracing system-related progression (Huang et al. 2007c), assessing the novel treatment strategies (Niethammer et al. 2017), and identifying the high-risk populations (Holtbernd et al. 2014).

PDRP scores are correlated with striatal dopamine transporter binding (Holtbernd et al. 2015) and subthalamic firing rate (Eidelberg 2009). Dopamine replacement therapy (Asanuma et al. 2006) and both subthalamic (Trošt et al. 2006) and pallidal deep brain stimulation reversed the PDRP expression (Fukuda et al. 2001). Levodopa treatment decreased metabolism in the sensorimotor cortex, cerebellar vermis, in the left globus pallidus, and ventral thalamus, while it increased metabolism in the prefrontal cortex (BA 10, 11), right cerebellar hemisphere, and precuneus (BA 7) in PD (Asanuma et al. 2006). A recent graph theory analysis revealed that the hypermetabolic basal ganglia is in the core of pathological brain network configuration associated with PD while the hypermetabolic cerebellum forms a separate subnetwork distinct with pons (Ko et al. 2018). These studies collectively suggest that the network modulation of PDRP may be a relevant secondary objective for preventive and/or disease modifying therapy trials.

While PDRP scores have been associated with overall disease progression and motor symptom severity, the second PC of SSM/PCA results has been associated with cognitive performance of PD patients (Huang et al. 2008). The PD-related cognitive pattern (PDCP) is therefore an archetypal characterization of PD with cognitive dysfunction. PDCP is spatially characterised by increased metabolic activity in the cerebellar cortex and dentate nuclei, and decreased activity in the medial frontal and parietal regions (Huang et al. 2007b). PDCP expression scores correlate with cognitive performance in verbal learning and executive function (Huang et al. 2007b; Meles et al. 2015) and that PDCP expression increases stepwise with cognitive decline (Mattis et al. 2016). Similar topographical brain network has been identified using resting-state fMRI and machine learning techniques (fPDRP) (Vo et al. 2017). Although both PDRP and PDCP expression levels (i.e., subject scores) have shown a linear correlation with symptom duration, the PDRP exhibited a faster progression compared to the PDCP over longitudinal follow-up period (Eidelberg 2009).

**Fig. 1:**
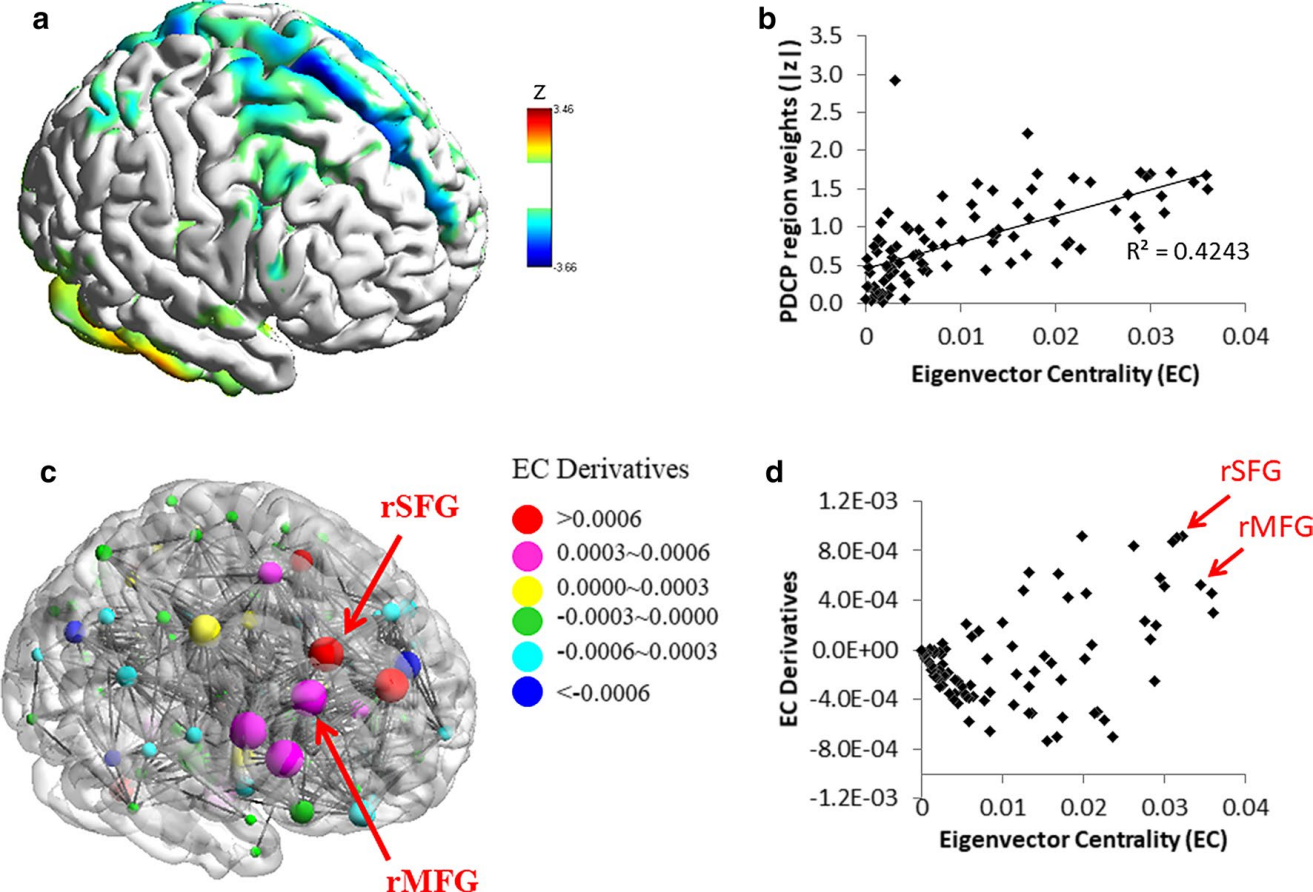
Topographic organization of metabolic networks associated with cognitive deficits in PD patients. **a** The previously documented PD-cognitive deficit-related metabolic pattern (PDCP) (Huang et al. 2007b). **b** Eigenvector Centrality (EC) values (i.e., relative nodal importance in information flow) analyzed by graph theory correlated with corresponding region weights of the PDCP. **c** The EC (radius of sphere) and EC derivatives within PDCP-relevant hubs (DEC; color of sphere) were visualized. DEC estimates the “sensitivity” of information flow to discrete perturbation against nodal centrality. The EC is correlated with the disease-related metabolic patterns’ region weights, and that the nodal sensitivity toward the metabolic network configuration can be estimated using a novel perturbation (simulation) method (Ko et al. 2018), which computes the DEC. **d** Bi-plot of the EC vs. DEC. The right middle frontal gyrus (rMFG, BA 8/9) and superior frontal gyrus (rSFG, BA 6/8) were identified with relatively high “importance” and “sensitivity” (red arrow). Adopted from Ko et al. (2014)

PDCP scores were correlated with caudate dopamine transporter binding (Niethammer et al. 2013), and they were correlated with levodopa-induced changes in cognitive performance in PD (Mattis et al. 2011). A recent graph theory analysis revealed that the right dorsolateral prefrontal cortex (DLPFC) is the most “sensitive” region that is associated with the PDCP configuration (Ko et al. 2014) (Fig. 1). Brain stimulation of both right superior and middle frontal gyri is thus expected to reverse PDCP expression in patients, by normalizing information flow within the PDCP network. These brain regions, often termed as DLPFC, are traditionally viewed as the core of PD cognitive dysfunction (Monchi et al. 2004; Polito et al. 2012) and thus chosen as the target in prior brain stimulation studies (Boggio et al. 2005; Sedláčková et al. 2009).

## Transcranial direct current stimulation (tDCS)

The electrical current (typically between 1 and 2 mA) of tDCS is applied via sponge electrodes (i.e., anode and cathode electrodes for increasing and decreasing neuronal excitability over the intended cortical target, respectively), with sizes varying from 15 to 80 cm^2^ (Horiba et al. 2019; Swank et al. 2018), to modulate neuronal membrane potential (Stagg et al. 2018). The stimulation time ranges from 7.5 to 30 min, with the most common stimulation protocol being 20 min long (Broeder et al. 2019a, b; Fernández-Lago et al. 2017; Horiba et al. 2019). The traditional model for the mechanisms underlying acute effects of tDCS is relatively straightforward. If anodal stimulation is applied, the neuronal membrane depolarizes—thus, with a less negative charge, the targeted neurons are more easily excitable, and spontaneous activity increases (Nitsche and Paulus 2000). If cathodal stimulation is applied, neuronal membranes hyperpolarize—thus increasing the necessary input to reach firing threshold. The target neurons are now less easily excitable and spontaneous activity decreases (Nitsche and Paulus 2000).

It should be noted that modeling studies suggest the effect of tDCS can vary according to the topography of cortical surface, i.e., the same anodal stimulation may depolarize or hyperpolarize depending on whether the target is in the gyri or sulci, which may explain the large inter-individual variability in tDCS responses (Rahman et al. 2013). In addition, the cognitive improvement seen after tDCS cannot simply be attributed to “more activity, better function,”—both depolarization and hyperpolarization may need to be interpreted as “neural noise,” the altered state of normal (or optimal) brain environment (De Berker et al. 2013). Computational neuroscience modeling studies on stochastic resonance suggests that some level of neural noise can indeed improve cognitive performance (McDonnell and Ward 2011). Due to this complexity, the relationship between tDCS polarity and the direction of excitability change (increase or decrease) is often determined based on the empirical observations of limited experiments (De Berker et al. 2013).

The after-effect is even more complicated. It depends on both glutamatergic and GABAergic synaptic activity as well as neuromodulators including dopamine, adenosine, serotonin, and acetylcholine (Stagg et al. 2018). The effect is not limited to the target regions, but also affects remote connected regions, as well as the feedback loop. While the underlying neuronal interactions are complicated, in general, the direction of the after-effect aligns with the acute effect, i.e., anodal stimulation typically increases neuronal excitability while cathodal stimulation decreases it (Stagg et al. 2018).

The majority of neurophysiological studies investigating the mechanisms underlying tDCS are based on motor cortex stimulation. (Broeder et al. 2019b; Horiba et al. 2019; Ishikuro et al. 2018). While this has greatly advanced our understanding, these findings are often non-transferable to other cortical regions. The prefrontal cortex is one of the most frequently targeted regions for cognitive improvement. In the absence of convenient neurophysiological examinations, such as motor-evoked potential size observation, combined tDCS-brain imaging studies can sufficiently validate the potential use of tDCS as a therapeutic tool and elucidate its underlying mechanisms (Ko et al. 2013). However, the biggest challenge is that the stimulation protocol is not optimized. Therapeutic application of tDCS takes several weeks to build up effects. It is therefore not feasible to design an optimization study with a full neuropsychological battery as an outcome variable. Since neurophysiological recordings are not available for prefrontal regions, researchers often have to choose a surrogate cognitive test to measure its acute effect, based on the assumption that a stimulation protocol that maximizes the acute effect may also be effective when applied for a longer term.

## Effects of tDCS on cognition networks

As of November 28, 2020, we have identified 7 clinical trials where tDCS effect on cognitive functions in PD have been investigated (PubMed search keywords: “Parkinson's disease”, “brain network”, “transcranial direct current stimulation”, “tDCS”, “functional brain imaging”, “cognitive”. In all studies, the DLPFC has been stimulated for 20–25 min at 2 mA intensity with the anode position on the targeted area. Three studies investigated the immediate effect of a single session tDCS Boggio et al. 2006; Lau et al. 2019; Pereira et al. 2013), while the effects of repeated sessions (10–16 sessions over 2–4 weeks) were investigated in four studies. Overall, five of seven studies showed a statistically significant cognitive improvement after the tDCS treatment on the DLPFC. One study reported no change, and the remaining reported worsened cognitive performance. The results of these studies are summarized in Table 1.

**Table 1:** List of studies exploring the effects of tDCS technique on cognitive deficits in PD

Author and year	Number of subjects	Age (y)	PD group	MMSE	Anti-parkinsonian drugs	Anatomical target	Intensity (mA)	Duration (min)	Number of sessions	Clinical Outcomes
Boggio et al. (2006)	18	61.1 ± 0.0	(MCI)	24.4 ± 3.1	12 h withdrawal	L-DLPFC M1 Sham	1 vs. 2	20	single session	Improved working memory after 2 mA tDCS on the L-DLPFC
Pereira et al. (2013)	16	61.5 ± 9.9	(MCI-NC)	27.7 ± 2.1	on	L-DLPFC L-TPC	2	20	single session	Improved phonemic fluency task
Doruk et al. (2014)	18	61.0 ± 8.0	(NC)	29.2 ± 0.30	on	L-DLPFC R-DLPFC sham	2	20	10 sessions in 2 weeks	Improved Trail Making Test B and executive function after the end of treatment and at 1-month follow-up
Biundo et al. (2015)	16	71.1 ± 5.8	MCI**	n/a	on	L-DLPFC sham	2	20	16 sessions in 4 weeks	Worsened attention/executive skill and memory index at the post-test, and improved memory index at the follow-up test
Manenti et al. (2016)	20	69.0 ± 0.0	MCI^†^-NC^‡^	n/a	on	L-DLPFC R-DLPFC sham	2	25	10 sessions in 2 weeks	Improved cognitive abilities after the end of treatment and at 3-month follow-up
Manenti et al. (2018)	22	64.0 ± 0.0	MCI^†^-NC^‡^	n/a	on	L-DLPFC sham	2	25	10 sessions in 2 weeks	Improved cognitive performances and reduction of depressive symptoms
Lau et al. (2019)	10	62.7 ± 6.6	(MCI)	26.2 ± 0.40	on	L-DLPFC Sham	2	20	single session	No change

*L-DLPFC* left dorsolateral prefrontal cortex, *R-DLPFC* right dorsolateral prefrontal cortex, *L-M1* left primary motor cortex, *L-TPC* left temporo-parietal cortex, *MCI* mild cognitive impairment, *MMSE* mini mental state examination, *NC* normal cognition

^†^Cognitive Rating Scale (PD-CRS) total score = 65–81

^‡^PD-CRS > 82

**Education = 8.9 ± 3.7, *n/a* not available. For studies where MCI status was not specifically defined, (MCI) and (NC) has been assigned according to the reported MMSE scores

Boggio et al. (2006) assessed working memory performance in 18 PD participants (12 men and 6 women, aged 45–71 years, mean age = 61.1 years, MMSE = 24.4 ± 3.1). Antiparkinsonian medications such as levodopa or dopaminergic agonist were held at least 12 h prior to the experiment. Patients performed 3-back working memory tasks during anodal tDCS applied on the primary motor cortex (M1) and the left DLPFC. The effects of different stimulation intensity (1 vs. 2 mA) was compared with sham stimulation. The left DLPFC stimulation (anodal; 2 mA) significantly improved working memory performance (i.e., increased number of correct responses, and decreased false alarms and response time), which was not observed when 1 mA tDCS was administered (Boggio et al. 2006).

In another crossover study, a single session of tDCS combined with fMRI was used for modulation of verbal fluency-related brain networks in 16 PD (7 men and 9 women, mean age = 61.5 ± 9.9 years, MMSE = 27.7 ± 2.1) (Pereira et al. 2013). To this end, the authors compared the effects of tDCS over the left DLPFC vs. the left temporo-parietal cortex (TPC). The left DLPFC stimulation (vs. the left TPC stimulation) improved verbal fluency and phonemic fluency task (i.e., increased number of words subjects produced in response to a letter). The left DLPFC stimulation group showed enhanced functional connectivity in task-related brain networks (i.e., medial frontal gyrus, posterior cingulate, bilateral parietal lobules, parahippocampus, caudate, cerebellum and inferior frontal gyrus regions) during a verbal fluency task. They also found younger patients had better responses to tDCS therapy than older patients, indicating that age should be considered as an important covariate in tDCS studies (Pereira et al. 2013). No significant correlations between phonemic or semantic fluency performance after DLPFC/TPC tDCS and dopaminergic daily doses was observed in PD patients (Pereira et al. 2013). Conversely, others have observed no improvement in visual working memory task or emotional go/no go performance after single-session tDCS over the left DLPFC in 10 PD patients (5 men and 5 women, aged 56–78 years, mean age = 62.7 ± 6.6, MMSE = 26.2 ± 0.40) (Lau et al. 2019).

Four studies assessed the effects of repeated tDCS sessions on cognitive function in PD (Biundo et al. 2015; Doruk et al. 2014; Manenti et al. 2018, 2016). All of these studies applied tDCS over the DLPFC at 2 mA intensity, with varying stimulation dose (10–16 sessions; 20–25 min per session).

Doruk et al. (2014) used a comprehensive test battery to evaluate immediate and long-term effects (e.g., 1-month follow-up) in 18 PD patients (12 men and 6 women, aged 40–71 years, mean age = 61.0 ± 8.0, MMSE = 29.2 ± 0.30). Ten sessions of anodal tDCS were completed (right DLPFC vs. left DLPFC vs. sham stimulation), and cognitive function, depressive symptoms, and motor function were measured in patients. Both active stimulation groups showed a significant improvement in executive function (i.e., Trail Making Tests A & B, Wisconsin Card Sorting Test, Probabilistic Classification Learning, Working Memory Test and Stroop Test) at the end of treatment and at 1-month follow-up. No significant effects were observed in mood (i.e., Beck Depression Inventory, the Hamilton Rating Scale for Depression, and the Hamilton Anxiety Scale) or motor symptoms (i.e., supination pronation, buttoning-up, finger tapping, walking time, purdue pegboard reaction time and motor part of Unified Parkinson’s Disease Rating Scale (UPDRS) (Doruk et al. 2014).

In a study on 16 PD patients with MCI (14 men and 2 women, mean age = 71.15 ± 5.8, education = 8.9 ± 3.7) (Biundo et al. 2015), tDCS was combined with a computerized cognitive training program. They found left DLPFC stimulation actually worsened attention/executive skills (assessed by written coding test) and memory (assessed by story learning test) immediately following the 4-week treatment sessions (16 sessions in total), although memory was improved at 4-month follow-up in PD patients with MCI compared to the sham stimulation. No significant changes were observed in motor symptoms as assessed by UPDRS-III.

When combined with physical therapy, anodal tDCS (10 sessions over 2 weeks; 2 mA; 25 min per session) over DLPFC (contralateral to side most affected by PD motor symptoms) improved patients’ cognitive function (assessed by Cognitive Rating Scale and verbal fluency) as compared to sham stimulation (Manenti et al. 2016) in 20 PD patients (11 men and 9 women; mean age = 69 years; PD-MCI, Cognitive Rating Scale (PD-CRS) total score = 65–81; PD-NC, PD-CRS > 82). This effect was sustained at 3-month follow-up after the last sessions. While patients also demonstrate improvement in motor and depressive symptoms, there was no significant difference between real vs. sham stimulation groups, indicating these changes were potentially induced by the physical therapy (Manenti et al. 2016).

A later study by Manenti et al. (2018) used 10 sessions of anodal tDCS, delivered over 2 weeks, combined with computerized cognitive training on PD patients with MCI as well as PD patients with normal cognition (12 men and 10 women, mean age = 64 years; PD-MCI, PD-CRS = 65–81; 10 PD-NC, PD-CRS > 82). They found a significant improvement in depressive symptoms (Beck Depression Inventory-II), cognitive performance in language (International Picture Naming Project, as well as phonemic and semantic verbal fluency tests), and attentional and executive function (Frontal Assessment Battery, Test of Attentional Performance, Stroop Test and Trail Making Test). This improvement remained at 3 month follow-up (Manenti et al. 2018).

## Challenges and future directions

The tDCS has been identified as an important prospective intervention for cognitive decline (Das et al. 2019), but few studies have sufficiently explored its potential for improving cognitive function in PD. The most considerable limitation in these existing studies has been small sample size—mostly not over 20 subjects. The small effect sizes exacerbate skepticism about the clinical utility of tDCS.

Previous neurophysiological studies and simulation studies have provided important insights on the potential underlying mechanisms of tDCS effects, yet its brain system-wide effects have not been fully addressed. For brain stimulation studies of neurodegenerative disorders, the stimulation target is typically determined based on abnormal metabolism, blood flow, task-induced hemodynamic response, or functional connectivity. Intuitively, researchers often position the electrodes to maximize the current flow in those targeted brain regions. However, previous FDG PET studies suggested that the metabolic changes induced by tDCS are not confined to the targeted area, rather more significant effects are found in remote brain regions (Im et al. 2019; Yoon et al. 2014). It should be noted that PD symptom expression is not confined to regional abnormality of a single brain region either, but it is associated with a circuit disruption (Ko et al. 2018). Therefore, the effects of tDCS must be investigated with the whole-brain as a network system. Our computer simulation study hinted the potential network re-organization of PDCP following targeted perturbation of the right DLPFC. Further clinical trials using tDCS combined with in vivo brain imaging techniques are warranted to further our understanding and treatment discovery for PD cognitive deficits.

The high inter-individual variability in tDCS response is another important hurdle for the clinical translation of tDCS technology. Combining neuroimaging methods with neurophysiological and psychological evaluations is a constructive method to examine why some individuals benefit vs. why others do not. This can further inform how to maximize tDCS benefits for each individual. Previous neuroimaging studies in healthy controls have clearly pointed out that the effect of tDCS is not limited to the targeted area (Mancuso et al. 2016). In a study with PD patients, different individuals utilized different brain regions while playing the same video game (Szturm et al. in press). To maximize tDCS effects, the target may need to be personalized to account for interindividual variability (Esmaeilpour et al. 2020). It is therefore not only advantageous, but necessary to combine neuroimaging techniques with current tDCS study designs to fully comprehend its systemic effects on the brain.

The virtually unlimited number of parameters that can be set for tDCS (e.g., stimulation location, intensity, duration, waveform types, and number of sessions) already makes it nearly impossible to empirically determine which protocol may result in the optimal outcome. Furthermore, optimal parameters may differ depending on what functions are targeted (e.g., motor vs. cognition vs. emotion) and who is being stimulated (inter-individual variability). A discussion must be initiated to develop a consortium and produce a personalized optimization guideline based on published literature, as well as unpublished raw data, so that a computational model can be developed to simulate and predict the outcome of the proposed tDCS strategy.

Out of 7 papers, three studies investigated the long-term impact (up to 3 months) of tDCS on cognitive deficits in PD (Biundo et al. 2015; Doruk et al. 2014; Manenti et al. 2016). It should be noted that the long-term effects of tDCS on brain molecular and cellular mechanisms can be different than immediate or short-term effects. The immediate effects are mostly mediated by direct changes in membrane potentials, such that anodal stimulation increases membrane potential, contributing to enhancing neuronal excitability, whilst the cathodal stimulation decreases membrane potential, leading to decreasing excitability across the anatomical target region (Pelletier and Cicchetti, 2015). The short-term after-effects may be mediated by neurotransmitters such as glutamate (Heimrath et al. 2020), gamma-aminobutyric acid (Nitsche et al. 2004), dopamine (Monte‐Silva et al. 2010), whereas long-term effects are thought to be correlated with alterations in gene expression (Kim et al. 2017) and protein synthesis (Cirillo et al. 2017; Ferreira et al. 2019). The effects of long-term tDCS have not been directly investigated in humans. More broadly, future clinical studies are also needed to examine the long-term effects of tDCS treatment on cognitive function and brain plasticity in PD.

The impact of medication use in tDCS effects is another important aspect which should be taken into consideration (McLaren et al. 2018). For instance, when different dose of levodopa (25, 100, and 200 mg) was co-administered in healthy individuals, the anodal tDCS effects (facilitatory) on the single pulse TMS-induced motor-evoked potentials (MEP; which is supposed to be increased after anodal tDCS) was abolished with 25 mg and 200 mg treatment while it was reversed to inhibition (i.e., TMS-induced MEP size is decreased) with 100 mg treatment (Monte‐Silva et al. 2010). The inhibitory effects of cathodal tDCS was also abolished with 20 mg and 200 mg of levodopa treatment while it was unaffected with 100 mg treatment (Monte‐Silva et al. 2010). This nonlinear relationship between levodopa dose and tDCS-induced plasticity further complicates the interpretation of the role of anti-parkinsonian medication on tDCS effects. In most tDCS studies (see Table 1), anti-parkinsonian medication was not withdrawn, and the medication interaction with tDCS therapy has never been systemically assessed within the context of PD cognition.

## Conclusion

Abnormal brain network expression in PD is relatively well-characterized. Neuroimaging studies have shed light on the neural mechanisms of PD cognitive deficits, and provided a potential therapeutic target for non-invasive brain stimulation techniques. A handful of research groups have tested the efficacy of tDCS for cognitive improvement in PD. Although most studies showed some potential, stimulation parameters must be further understood and optimized to make it useful in clinical settings. The use of neuroimaging combined with tDCS is warranted and may aid both target identification and outcome assessment for future tDCS trials.

## Data Availability

Not applicable.

## Abbreviations

AD: Alzheimer’s disease
DBS: Deep brain stimulation
DLPFC: Dorsolateral prefrontal cortex
fMRI: Functional magnetic resonance imaging
M1: Primary motor cortex
MCI: Mild cognitive impairment
MMSE: Mini mental state examination
PCA: Principal component analysis
PD: Parkinson’s disease
PDCP: PD-related cognitive pattern
PDRP: PD-related metabolic covariance pattern
PET: Positron emission tomography
SMA: Supplementary motor area
SSM: Scaled subprofile modeling
tDCS: Transcranial direct currention stimulation
TMS: Transcranial magnetic stimulation
TPC: Temporo-parietal cortex
UPDRS: Unified Parkinson’s Disease Rating Scale
VAF: Variances accounted
